# The Effect of Chemical Chaperones on the Assembly and Stability of HIV-1 Capsid Protein

**DOI:** 10.1371/journal.pone.0060867

**Published:** 2013-04-05

**Authors:** Ayala Lampel, Yaron Bram, Michal Levy-Sakin, Eran Bacharach, Ehud Gazit

**Affiliations:** 1 Department of Molecular Microbiology and Biotechnology, Tel Aviv University, Tel Aviv, Israel; 2 Department of Cell Research and Immunology, Tel Aviv University, Tel Aviv, Israel; Queensland Institute of Medical Research, Australia

## Abstract

Chemical chaperones are small organic molecules which accumulate in a broad range of organisms in various tissues under different stress conditions and assist in the maintenance of a correct proteostasis under denaturating environments. The effect of chemical chaperones on protein folding and aggregation has been extensively studied and is generally considered to be mediated through non-specific interactions. However, the precise mechanism of action remains elusive. Protein self-assembly is a key event in both native and pathological states, ranging from microtubules and actin filaments formation to toxic amyloids appearance in degenerative disorders, such as Alzheimer's and Parkinson's diseases. Another pathological event, in which protein assembly cascade is a fundamental process, is the formation of virus particles. In the late stage of the virus life cycle, capsid proteins self-assemble into highly-ordered cores, which encapsulate the viral genome, consequently protect genome integrity and mediate infectivity. In this study, we examined the effect of different groups of chemical chaperones on viral capsid assembly *in vitro*, focusing on HIV-1 capsid protein as a system model. We found that while polyols and sugars markedly inhibited capsid assembly, methylamines dramatically enhanced the assembly rate. Moreover, chemical chaperones that inhibited capsid core formation, also stabilized capsid structure under thermal denaturation. Correspondingly, trimethylamine *N*-oxide, which facilitated formation of high-order assemblies, clearly destabilized capsid structure under similar conditions. In contrast to the prevailing hypothesis suggesting that chemical chaperones affect proteins through preferential exclusion, the observed dual effects imply that different chaperones modify capsid assembly and stability through different mechanisms. Furthermore, our results indicate a correlation between the folding state of capsid to its tendency to assemble into highly-ordered structures.

## Introduction

Self-assembly of single molecules into ordered supra-molecular structures is a fundamental process in biology and chemistry [1]–[4]. Nano-scale biological structures, such as microtubules, actin filaments and bacterial flagella are built through a coordinated association of folded protein subunits using a defined set of intermolecular interactions. Targeting these essential controlled assembly events holds a therapeutic potential toward pathological conditions; for example, drugs that target the organization of the cytoskeleton are used as anti-cancer agents [5].

An important, naturally-occurring, self-assembly process with a major pathological outcome is the buildup of virus particles (virions), including the formation of highly- ordered cores, which encapsidate and protect the viral genome [6]. An extensively studied model for such process is the formation of the human immunodeficiency virus (HIV) particle and core structure by the viral capsid (CA) protein [7]. CA is synthesized as one of the domains of the Gag precursor and multimerization of the latter is a key event in the formation of an immature, noninfectious HIV particle. Gag multimerization is mediated in part by intermolecular interactions of CA domains. After a maturation step that involves the proteolytic cleavage of Gag, CA is released from its precursor and self-assembles into a conical core (capsid) structure within the mature virion. Such maturation process renders the virion infectious [8]–[10]. Altogether, CA plays a critical role in two assembly events during HIV replication cycle [8], [11], and thus presents an attractive therapeutic target for viral inhibition [12].

Considerable efforts have been put into developing *in vitro* assembly systems, in which purified HIV structural domains can be induced to polymerize into biologically relevant supra-molecular structures [13]–[22]. CA can be induced to polymerize into tubular polymers in the presence of RNA, as part of the CA-nucleocapsid (NC) fusion complex [14]. CA alone (or CA-NC) can form tubes and cones, but only at very high ionic strength [15]–[20]. Cryo-electron microscopy (CryoEM) images revealed that these structures composed of helical arrays of CA hexamers [22], similar to the structural organization of native mature capsids [23]. Furthermore, mutational analyses demonstrated that *in vitro* assemblies of CA cylinders share structural similarities with the *in vivo* mature capsid assemblies [16], [24]. Therefore, CA polymers, assembled *in vitro*, are considered to represent authentic assemblies of the viral core.

CA assembly *in vitro* is highly sensitive to a variety of physicochemical conditions including temperature, ionic strength and pH [14], [18]–[19], [21], [25]–[29]. In addition, the presence of crowding agents, which mimic the dense milieu inside the virion, triggers the *in vitro* assembly of CA [30]. Crowding agents, as well as other cellular solutes, are commonly assumed to affect protein stability and aggregation through preferential exclusion from the protein surface [31]–[32], which increases the free energy of the unfolded state of the protein. An additional group of solutes, considered to affect proteins in the same manner, is cellular osmolytes [33]–[34], small natural organic molecules that contribute to the maintenance of cell volume [35]. In addition, osmolytes have a role in stabilization of protein structure under different stress conditions [36]–[41] and therefore were termed ‘chemical chaperones’ [37], due to the similarity in function to molecular chaperones.

Chemical chaperones, like the molecular ones, may accumulate under stress conditions in various organisms. In such conditions, these osmolytes may induce protein folding, stabilization [36]–[40], oligomerization, reduce aggregation [42] and prevent protein mislocalization [43]. Usually, they affect protein stability at high millimolar to molar range of concentrations. As has been extensively reviewed previously by Yancey et al., [36] these solutes fall into a few major chemical categories: small carbohydrates including sugars (e.g. trehalose), polyols (e.g. glycerol); amino acids (e.g. taurine) and methylamines [e.g. trimethylamine *N*-oxide (TMAO)] and methylsulfonium solutes including dimethylsulfonopropionate (DMSP). According to their effect on protein stability, the use of chemical chaperones was suggested as a therapeutic tool in case of protein conformational-associated diseases [42], [44]–[45].

While the effect of chemical chaperones on the aggregation of misfolded proteins has been extensively studied [42], [46]–[51], the effect of chemical chaperones on virus self-assembly processes is insufficiently investigated. Here we examined the effect of four major groups of naturally-occurring chemical chaperones on the assembly, structure and stability of HIV CA protein. Polyols and sugars were found to stabilize CA structure under thermal stress and markedly inhibited CA assembly. In contrast, different methylamines accelerated the assembly of CA and TMAO was also found to induce CA unfolding. Hence, these results imply a correlation between CA structural stability and its tendency to self-assemble. Moreover, our data suggest that interference with HIV CA assembly by chemical chaperones may serve as a strategy for the development of anti-viral therapy.

## Results

### Effect of chemical chaperones on the *in vitro* assembly of HIV-1 CA protein

**Figure 1:**
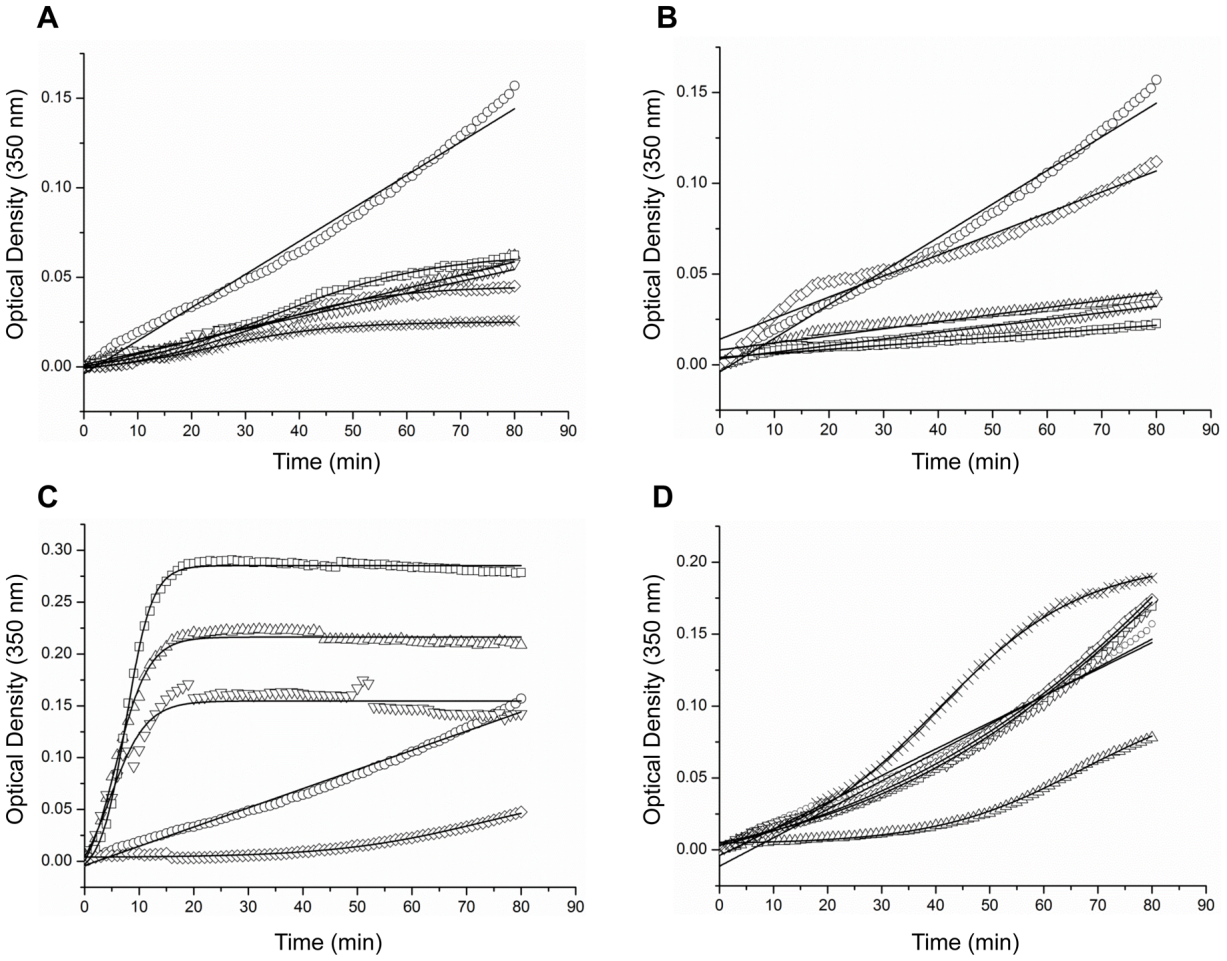
Effect of chemical chaperones on the kinetics of HIV-1 CA assembly. *In vitro* CA assembly assay was performed in 50 mM Na_2_HPO_4_ buffer (pH 8.0) containing CA (38 µM) and NaCl (1.5 M) in the absence (circles) or presence of 0.5 M of (**A**) polyols: sorbitol (triangles); adonitol (inverted triangles); meso-erythritol (squares); glycerol (diamonds) or ethylene glycol (crosses), (**B**) sugars: maltose (triangles); arabinose (inverted triangles); mannose (squares) and trehalose (diamonds), (**C)** methylamines: TMAO (triangles); betaine (inverted triangles); sarcosine (squares) and choline chloride (diamonds), (**D**) amino acids: Trp (triangles); Tyr (inverted triangles); Leu (squares); Val (diamonds) and taurine (crosses) (1 mM for tyrosine and 5 mM for the rest of the amino acids). Turbidity was monitored at wavelength of 350 nm, using spectrophotometer.

To examine the effect of different chemical chaperones on the assembly of HIV-1 CA, we applied an *in vitro* polymerization kinetic assay [19], in which a high salt concentration is used to trigger the assembly of CA. For that purpose, recombinant full-length CA was expressed and purified using selective precipitation followed by anion exchange chromatography [19]. The purified protein was rapidly diluted in the assay buffer either in absence or presence of a variety of polyols, sugars, methylamines (0.5 M) or amino acids (1 mM for Tyr and 5 mM for the additional amino acids); and the formation of CA assemblies was monitored over time by measuring the turbidity of the sample (Fig. 1). The time-dependent formation of light-scattering CA polymers fitted the Boltzmann equation (Equation 1). Assembly reactions of CA in the absence or presence of different polyols or sugars yielded a moderate increase in the turbidity of the samples over the measured time, which did not reach a plateau. Therefore, for these curves we applied a linear fit. We should stress that initial rate of assembly, as was determined by linear fit of the changes in the optical density for the approximate linear part of the assembly curves, was calculated for all the reactions (Table 1). This analysis demonstrated that the presence of either polyols (Fig. 1A, Table 1), or sugars (Fig. 1B, Table 1) inhibited CA assembly when compared to the control sample. The most dramatic decrease in the initial assembly rate was achieved by mannose.

**Table 1:** Values of kinetic parameters of CA assembly either in the absence or presence of different chemical chaperones.

Sample	Fitting model	t_50_ a(min)	Linear rate^b^(OD/min)
Control	Linear	>40	0.0017
Sorbitol	Linear	>40	0.0007
Adonitol	Linear	>40	0.0007
meso-Erythritol	Boltzman	35.3±0.41	0.0007
Glycerol	Boltzman	30.3±0.41	0.0008
Ethylene glycol	Boltzman	23.7±0.63	0.0005
Maltose	Linear	>40	0.001
			0.0003
Arabinose	Linear	>40	0.001
			0.0004
Mannose	Linear	>40	0.0008
			0.0002
Trehalose	Linear	>40	0.0027
			0.0007
Trp	Boltzman	67.0±1.08	0.0003
Tyr	Linear	>40	0.0014
Leu	Boltzman	90.1±4.95	0.0012
Val	Boltzman	120.7±9.86	0.0014
Taurine	Boltzman	40.4±0.18	0.0014
TMAO	Boltzman	5.7±0.32	0.0163
Betaine	Boltzman	4.6±1.28	0.0139
Sarcosine	Boltzman	7.6±0.08	0.0219
Choline chloride	Boltzman	72.9±2.81	0.0005

a t_50_ is the time at which the optical density (OD) is equal to one-half the optical density extrapolated at infinite time. The values given were obtained by fitting the data to equation 1. The fitting errors are indicated. For linear-fitted curves, the t_50_ values cannot be calculated but rather estimated to be >40 min, since the maximal t value is 80 min.

b The linear polymerization rate is the average increase in optical density per minute for the approximate linear part of the polymerization curve. Two values of assembly rate are indicated for curves with two phases of linear increase.

While some of the polyols and sugars significantly inhibited CA polymerization (Fig. 1A, B), three methylamines: TMAO, betaine and sarcosine, strongly accelerated the rate of CA assembly (Fig. 1C, Table 1). Specifically, the presence of sarcosine accelerated the rate of assembly by about 13-fold. In the presence of TMAO, betaine or sarcosine, CA polymerization reached a plateau after 15 min incubation, whereas in their absence, the polymerization process was still in the linear phase even after 80 min (Fig. 1C). Choline chloride, in contrast, mildly inhibited CA polymerization (Fig. 1C, Table 1).

When the effect of amino acids was tested in this assay, most of the compounds (Tyr, Leu, Val and taurine) showed no effect on CA assembly (Fig. 1D, Table 1). In contrast, the presence of Trp moderately inhibited the assembly of CA. It was previously shown that a Trp molecule positioned at the dimerization interface plays a critical role in the assembly of CA [52]. Accordingly, the inhibition of CA assembly by the free Trp may represent the masking of Trp184, positioned at the dimerization interface of CA (Fig. 1D).

**Figure 2:**
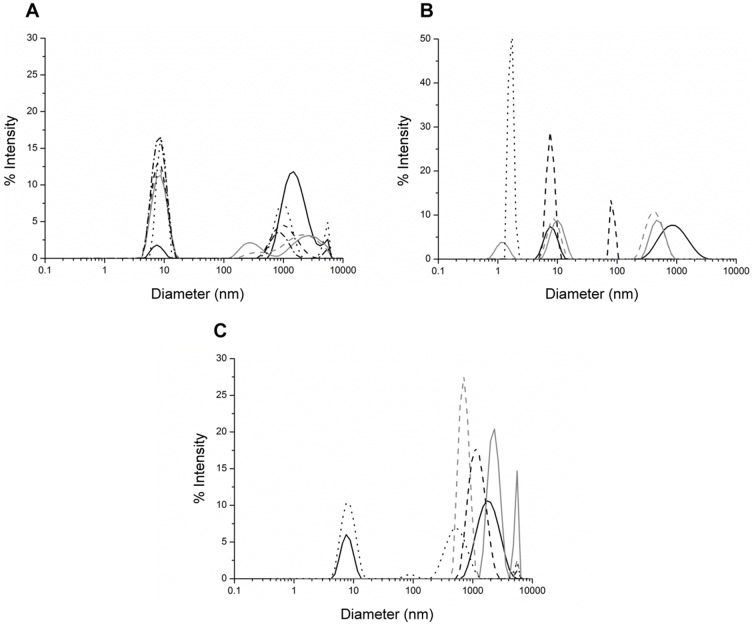
Dynamic light scattering measurements of CA assemblies. Assembly of CA was carried out using the same conditions as in Fig. 1. The hydrodynamic radius (nm) of CA particles was measured after 30 min incubation in the absence (black solid line) or presence of 0.5 M of (**A**) polyols: sorbitol (grey solid line); adonitol (black dashed line); meso-erythritol (grey dashed line); glycerol (dotted line) or ethylene glycol (dashed-dotted line), 5 min incubation in the absence (black solid line) or presence of (**B**) sugars: maltose (grey solid line); arabinose (black dashed line); trehalose (grey dashed line) or mannose (dotted line), and 10 min incubation in the absence (black solid line) or presence of (**C**) methylamines: TMAO (grey solid line); betaine (black dashed line); sarcosine (grey dashed line) or choline chloride (dotted line).

Although useful in the measurement of the kinetics of CA assembly, turbidity does not provide information regarding size distribution of the formed assemblies. The assembly of CA *in vitro* is initiated with the formation of small oligomers, which quickly associate to form larger structures [15]. Thus, we measured the effect of chemical chaprones on the formation of the different assemblies by dynamic light scattering (DLS). DLS is generally being used to assess the hydrodynamic radii of spherical particles. Since CA assembles *in vitro* into tubular and conical structures [15], the exact hydrodynamic diameter of these particles cannot be simply calculated using this technique; yet, DLS tracks fluctuations in size distribution of different populations of particles in solution and therefore can be used to monitor assembly reactions. In preliminary experiments in which we monitored CA assembly (using the same conditions as above), at zero time, two populations of assemblies were observed; one distributed around 8 nm and the other around 500 nm. In a previously reported study performed by Ehrlich et al., [25] CA assemblies were measured using both DLS and static light scattering (SLS). Particle population with a diameter of 2–3 nm was characterized as CA dimers. Therefore, we assumed that the 8 nm population represents CA oligomers. Characteristically to assembly processes, as the incubation time increased, the population of oligomers decreased whereas the population of high-order assemblies, which shifted to distribute around 1,000 nm, increased (data not shown). As presented in Fig. 2, DLS analysis revealed that after 30 min incubation, the presence of polyols (0.5 M) (Fig. 2A) resulted in the formation of small assemblies (distributed around ∼8 nm) rather than the larger assemblies (with hydrodynamic diameters peaking at >1,000 nm) which formed in the absence of these chaperones. In addition, the formation of large-diameter CA particles was reduced in the presence of sugars (0.5 M) after 5 min incubation (Fig. 2B), where populations of particles with a diameter of <2 nm were detected in the presence of maltose or trehalose. Due to the physical limitation of the DLS, the diameter of maltose and trehalose molecules (360 D and 378 D, respectively) cannot be directly measured; hence, we cannot determine the origin of the <8 nm peak. However, based on the findings of Ehrlich et al., [25] and the presence of a single 1–2 nm population in the CA/trehalose sample, we suggest that this population represents CA monomers. In contrast, the presence of TMAO, betaine or sarcosine (0.5 M) facilitated the formation of large assemblies (diameters distributed around 1,000 nm), after 10 min incubation (Fig. 2C). Yet, choline chloride (0.5 M) inhibited the formation of these large CA particles (Fig. 2C). Overall, the DLS results correspond with the turbidity measurements, further demonstrating that polyols, sugars and choline chloride inhibit the formation of high-order CA assemblies while the methylamines TMAO, betaine and sarcosine facilitate the formation of these particles (Fig. 1).

### Ultrastructural analysis of mature-like particles in the presence of chemical chaperones

**Figure 3:**
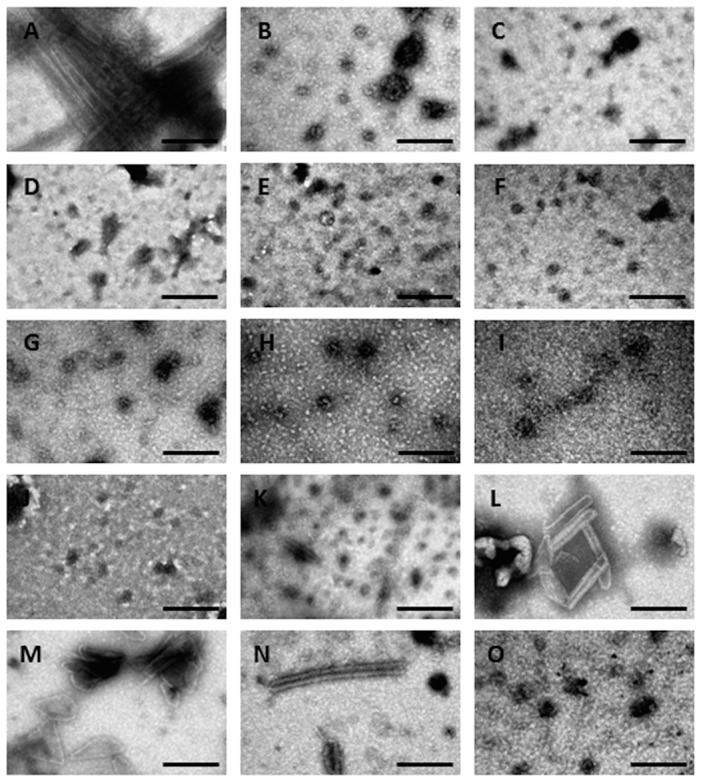
TEM micrographs of mature-like CA particles. CA particles formed after 1 h incubation in the absence (A) or presence of 0.5 M of sorbitol (B), adonitol (C), meso-erythritol (D), glycerol (E), ethylene glycol (F), maltose (G), arabinose (H), mannose (I) or trehalose (J). CA structures formed after 30 min incubation in the absence (K) or presence of 0.5 M of TMAO (L), betaine (M), sarcosine (N) or choline chloride (O). Scale bar: 200 nm.

To reveal the effect of chemical chaperones on the ultrastructural properties of CA assemblies, CA association was induced as described above, in the presence of the indicated chemical chaperones (0.5 M). After 1 h incubation (except for methylamines, for which incubation time was reduced by half - see below), the samples were analyzed using transmission electron microscopy (TEM). In the absence of chaperones, CA assembled into long hollow tubes with an external diameter of about 50 nm (Fig. 3A). In the presence of each of the five examined polyols, no tubes were detected; instead, small amorphous globular structures were observed (Fig. 3B–F). In addition, globular structures were identified in the presence of the indicated sugars (Fig. 3G–J). In contrast, large amounts of assemblies were observed in the presence of TMAO, betaine and sarcosine, which resulted in an excessive load that damaged the carbon coating of the grid (data not shown). Therefore, a shorter period of incubation (30 min) was employed for these three methylamines, resulting in the formation of clear hollow tubes (Fig. 3L–N). Remarkably, when the incubation time of the control sample (in the absence of chaperones) was reduced accordingly, only few assemblies were observed (Fig. 3K), further emphasizing the enhancement effect of the above methylamines on the assembly of CA. Moreover, conical structures that resemble authentic mature HIV capsids were detected in the presence of betaine (Fig. 3M). CA structures that were formed in the presence of choline chloride differed from the other methylamines since only amorphous structures were observed in its presence (Fig. 3O). Overall, the TEM analysis correlates well with the quantitative analyses (Fig. 1 and 2).

### Chemical chaperones affect CA structural stability

**Figure 4:**
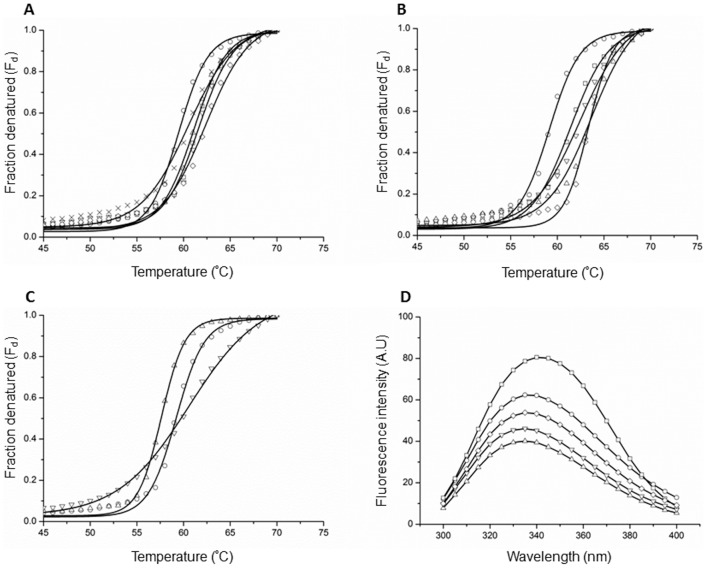
Effect of chemical chaperones on the thermal stability of CA. Thermal stability of CA (10 µM) in 50 mM Na_2_HPO_4_ buffer (pH 8.0) was measured using CD spectroscopy. The fraction of denatured protein (F_d_) was obtained following the changes in ellipticity at 222 nm as a function of temperature, in absence (circles) or presence of 1 M of (**A**) polyols: sorbitol (triangles); adonitol (inverted triangles); meso-erythritol (squares); glycerol (diamonds) or ethylene glycol (crosses), (**B**) sugars: maltose (triangles); arabinose (inverted triangles); mannose (squares) and trehalose (diamonds) or (**C**) methylamines: TMAO (triangles) or choline chloride (inverted triangles). (**D**) Trp intrinsic fluorescence emission spectra of CA (1 µM) in 50 mM Na_2_HPO_4_ buffer (pH 8.0), incubated at 60°C for 5 min, in absence (circles) or presence of 1 M of different chemical chaperones: adonitol (triangles); maltose (inverted triangles); TMAO (squares) or choline chloride (diamonds).

Polyols, sugars and methylamines were shown to induce protein folding and stabilization under stress conditions [36]. To determine whether the chemical chaperones affect CA assembly by altering the conformation or stability of CA, thermal stability was evaluated using circular dichroism (CD) spectroscopy, at a relatively low protein concentration (10 µM). Under these conditions the protein is mostly in monomeric and dimeric forms [52]. The CD analysis of CA revealed a predominant α-helical conformation with two negative peaks at 208 and 222 nm (data not shown), consistent with the reported X-ray crystallographic structure [52]. Thermal unfolding transition midpoints of CA in the absence or presence of chemical chaperones (1 M) were determined by monitoring changes in the ellipticity at 222 nm. Co-incubation of CA with each of the polyols increased the midpoint of folding transition (T_m_) by 1.0–2.9°C (Fig. 4A, Table 2), suggesting an increase in the stability of CA conformation. In a similar manner, the presence of sugars elevated the T_m_ of CA, where trehalose and maltose showed the maximal increase in T_m_ (4.1°C and 4.4°C, respectively; Fig. 4B, Table 2). Interestingly, the presence of TMAO decreased the T_m_ of CA by 1.6°C (Fig. 4C, Table 2), which may hint for destabilization of CA structure. In contrast, the presence of choline chloride resulted in an increase (1.4°C) in the T_m_ of CA. Notably, the presence of choline chloride resulted in a unique pattern of denatured/folded ratio of CA: at temperatures lower than 57°C, there was an increase in the fraction of denatured protein, whereas at temperatures higher than 57°C, the presence of choline chloride decreased the fraction of denatured CA (Fig. 4C), implying for stabilization of CA folding only at high temperatures. Due to the high absorbance measured in the presence of betaine or sarcosine (data not shown), we could not monitor their effect on the thermal stability of CA using CD.

**Table 2:** Changes in the thermal stability (°C) of CA in the absence or presence of chemical chaperones.

Sample	T_m_ (°C)^a^
Control polyols	59.5±0.09
Sorbitol	61.1±0.13
Adonitol	61.6±0.15
meso-Erythritol	61.4±0.13
Glycerol	62.4±0.19
Ethylene glycol	60.5±0.21
Control sugars	59.2±0.12
Maltose	63.6±0.28
Mannose	62.4±0.21
Arabinose	61.4±0.13
Trehalose	63.3±0.11
Control methylamines	59.2±0.09
TMAO	57.6±0.06
Choline chloride	60.6±0.18

a T_m_ refers to the folding transition midpoint (T_m_) of CA in the absence or presence of chemical chaperones. The presented values were obtained by fitting the data to equation 2 (see materials and methods). Fitting errors are indicated.

We used intrinsic Trp fluorescence spectroscopy as a complementary method to further validate the effect of the chaperones on CA folding. When incubated at 25°C, at a final concentration of 1 µM, CA showed a maximum emission wavelength of about 335 nm. After 5 min incubation at 60°C, a red-shift to 338 nm was observed. This shift most likely indicates protein denaturation process, since the addition of molar concentrations of urea similarly resulted in a red-shift of the CA emission spectra (data not shown). The emission spectra of CA were recorded after 5 min incubation at 60°C, in the absence or presence of chemical chaperones (1 M). As demonstrated in Fig. 4D, the maximum emission intensity of CA was increased by ∼20% in the presence of TMAO, when compared to its intensity in the absence of the chaperone. In addition, the maximum emission wavelength was red-shifted (from ∼338 to ∼341 nm). In the presence of choline chloride, adonitol or maltose, the maximum emission intensity was reduced by 13%, 35% and 26%, respectively. No red-shift was observed with these compounds. Accordingly, and in agreement with the CD results, while TMAO induced unfolding and destabilization of CA, choline chloride, adonitol and maltose stabilized CA structure and induced folding under thermal stress.

Taken together, we observed an interesting behavior, molecules that hindered CA assembly (shown by turbidity assay, DLS and EM analyses), attenuated CA denaturation under heating. In a similar manner, TMAO which induced CA association also facilitated CA denaturation, as revealed by CD and the intrinsic fluorescence analyses.

## Discussion

In the current work, we studied the effect of different groups of chemical chaperones on stability and assembly of HIV-1 CA. Chemical chaperones are known to have profound effects on protein folding and self-assembly; among others, osmolytes have been reported to modify the assembly cascade of amyloidal peptides, a hallmark incident in several degenerative diseases. While different chemical chaperones inhibited this process [49], [53]–[54], others accelerated such amyloid formation [53], [55]. Another important self-assembly process with serious healthcare implications is CA assembly, a key event in HIV-1 maturation and infectivity [8], [11]. Consequently, CA has become an attractive target for antiviral drug design in recent years [12]; yet, the effect of chemical chaperones on CA assembly has not been extensively examined.

We examined the effect of chemical chaperones on CA stability and assembly *in vitro* using different biophysical methods and ultrastructural analysis. It is commonly assumed that chemical chaperones facilitate protein folding, structure stability and compact conformation. As expected, the methylamine choline chloride and the examined polyols and sugars stabilized CA structure. However, TMAO, a well-known protein stabilizer [38], had an opposite destabilizing effect on CA structure. These results were inferred from far-UV CD measurements, showing an increase (by choline chloride, polyols or sugars) or decrease (by TMAO) in CA T_m_. Trp intrinsic fluorescence measurements confirmed previous results demonstrating reduction (by choline chloride, adonitol or maltose) or enhancement (by TMAO) in the maximum emission intensity of CA upon heating. The emission spectra of CA was red-shifted in the presence of TMAO, indicating an exposure of the core Trp residues to the solvent due to CA unfolding. Notably, the different effects that were observed by the tested chaperones on CA structure were merely evident on the metastable state of CA (under physiological conditions, their effect was insignificant), supporting the biocompatibility attributed to osmolytes, due to their negligible effect on the structure of native proteins [38].

In accordance to the structure stability effects that were observed, the self-assembly of CA was differentially affected by the various chemical chaperones. The examined polyols and sugars strongly reduced the rate of CA assembly and the methylamine choline chloride mildly inhibited this process. In contrast, the methylamines TMAO, betaine and sarcosine markedly accelerated the self-assembly of CA. The tested amino acids did not affect CA assembly, except for Trp that attenuated the kinetics of this process. This effect may reflect the interference of the free Trp with the mutual docking of neighboring Trp184, positioned at the dimerization interface of CA, critical for the assembly of the protein [52]. DLS and TEM analyses were utilized to provide more specific measurements regarding the size and morphology of CA assemblies. In the absence of chemical chaperones, CA assembled into high-molecular weight conformers with tubular morphology. In the presence of choline chloride, polyols or sugars, CA formed mostly low-molecular weight assemblies with spherical appearance. The acceleration of CA assembly by TMAO, betaine or sarcosine was demonstrated by the rapid shift of CA into tubes and cones assemblies, resembling structures formed in the control sample after longer incubation time.

The ability of chemical chaperones to stabilize proteins against denaturing stresses originates from the unfavorable interaction of the molecules with the protein backbone. Unfavorable interactions between a solvent component (the chemical chaperones) and a protein functional group (CA backbone) are traditionally classified as solvophobic. Preferential exclusion between the chemical chaperone and the protein implies net unfavorable (solvophobic) interactions that yield an increase in the Gibbs energy of the protein species [56].

**Figure 5:**
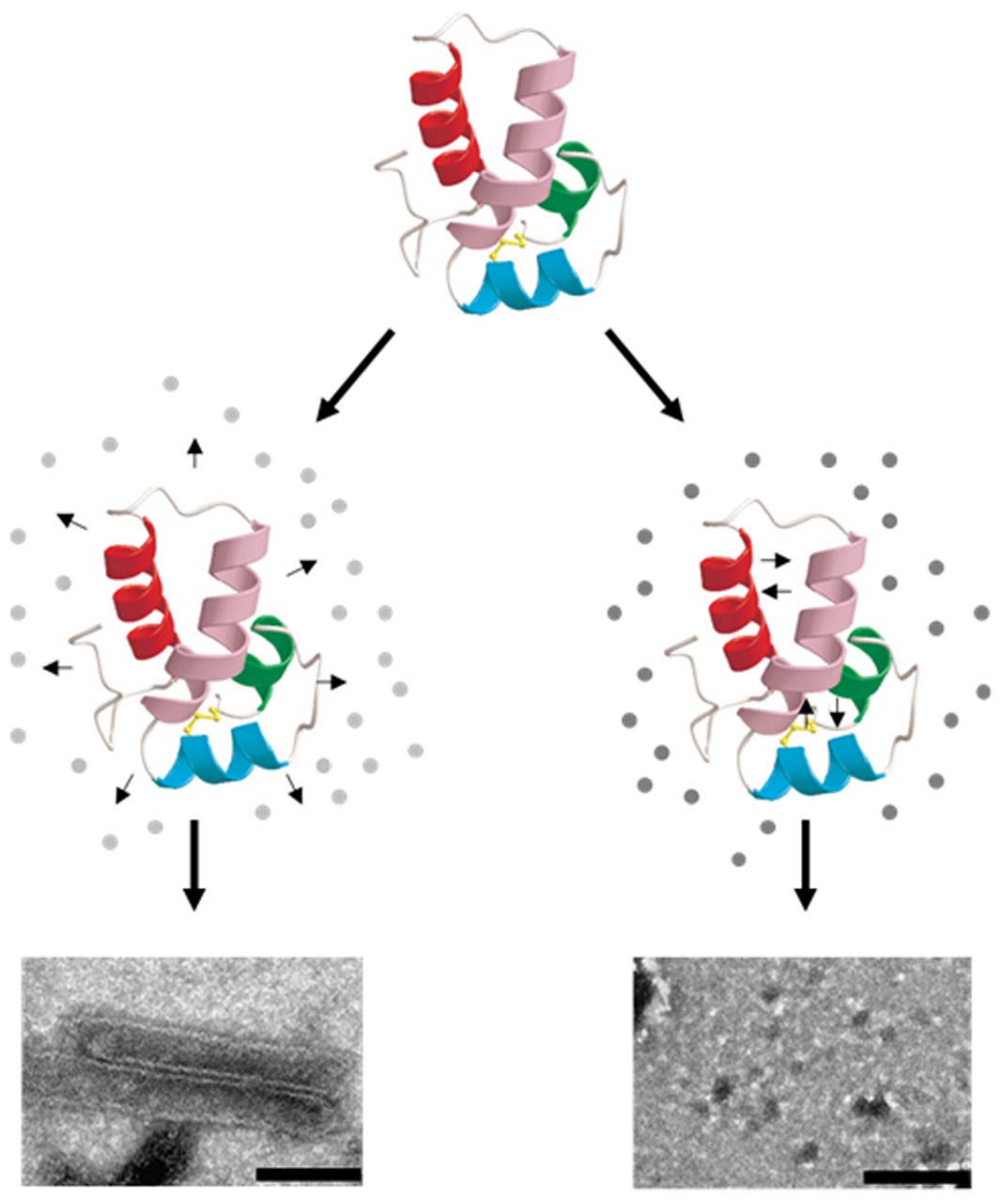
Schematic model for the effect of different chemical chaperones groups on CA stability and assembly. The presence of polyols or sugars (dark grey circles) induces compactization of CA structure and inhibits the formation of high-order CA structures. In contrast, the presence of the methylamines TMAO, betaine or sarcosine (light grey circles) destabilizes CA structure and thus promoting CA-CA interactions, resulting in the formation of CA cylinders. Structure of HIV-1 CA (151–231) created using PDB 1A8O [52] represents full-length CA protein. The scale bars are 100 nm (left image) and 200 nm (right image).

We suggest that the partially unfolded state of CA, induced either by heating (thermal denaturation experiment) or by high ionic strength (used to trigger CA assembly), results in the exposure of hydrophobic patches that promote intermolecular interactions and CA assembly. According to this model (Fig. 5), polyols and sugars stabilize the lower-energy-native-CA conformer (the compact folded state of CA), reducing the levels of partially unfolded CA protein and thus impair CA self-assembly. Therefore, we suggest that polyols and sugars affect CA assembly by compactization and stabilization of CA structure; in contrast, the methylamines TMAO, betaine and sarcosine destabilize CA structure, hence promoting CA-CA interactions. The contrary effect of the different groups of chemical chaperones on the stability and assembly of CA may be explained by a different capacity of each group to form hydrogen bonds.

Taken together, these results corroborate the assumption that protein self-association proceeds via a partially exposed hydrophobic core in a molten globule-like species as was proposed for the process of amyloids self-assembly [57]. Thus, destabilization of CA structure facilitates the formation of higher-ordered assemblies, as was suggested in the process of domain-swapped dimerization of CA C-terminal domain during the assembly of the immature particle [58].

To conclude, we have found that various chemical chaperones have opposing effects on HIV-1 CA assembly and stability. These results may provide additional tools for *in vitro* manipulation of capsids of HIV, and likely of other viruses. In addition, the notion that partial unfolding of CA facilitates its self-assembly provides a conceptual framework for the design of a new class of anti-viral drugs that stabilize a rigid conformation of this protein, thus hinder virus assembly.

## Materials and Methods

### Protein expression and purification

Expression vector of HIV-1 full length CA protein was kindly provided by W. Sundquist (University of Utah). HIV-1 CA was expressed in BL21 (DE3) *Escherichia coli* cells, and purified as previously described [19]. Briefly, bacteria culture was grown to an *A*_600_ of 0.8 at 37°C and induced with 0.4 mM IPTG (isopropyl-β-D-thiogalactopyranoside). After 4 h, the cells were collected by centrifugation and stored at −80°C. Cells were lysed by 12.5 mg/ml of lysozyme in 50 mM Tris-HCl (pH 8.0) 5 mM β-mercaptoethanol and precipitated with 25% of saturated ammonium sulfate. The crude CA was bound to 8-ml Q-Sepharose column (Amersham Pharmacia Biotech) and eluted with 25 mM Tris-Cl (pH 8.1) and 75 mM NaCl.

### Kinetic analysis of CA assembly

Purified CA was dialyzed against 50 mM Na_2_HPO_4_ at pH 8.0, assembly was triggered by the addition of 50 mM Na_2_HPO_4_-4M NaCl at pH 8.0 [19]. Assembly reactions were carried out at a final NaCl concentration of 1.5 M, and final protein concentration of 38 µM. CA was assembled in the absence or presence of 0.5 M of different methylamines (TMAO, Fluka; betaine, sarcosine or choline chloride, Sigma-Aldrich), polyols (sorbitol, adonitol or ethylene glycol, Sigma-Aldrich; glycerol, Biolab; meso-erythritol, Acros) and sugars (trehalose, Across; maltose, arabinose or mannose, Sigma-Aldrich), 5 mM of amino acids (tryptophan, leucine, valine or taurine, Sigma-Aldrich) or 1 mM of tyrosine (Sigma-Aldrich). The increase in optical density was monitored over 80 min in 1 min intervals, using a Biotek® Synergy™ spectrometer. Each data point represents an average of a triplicate. For most assembly reactions (CA in the presence of meso-erythritol, glycerol, ethylene glycol, Trp, Leu, Val, Taurine, TMAO, betaine, sarcosine or choline chloride) the time-dependent increase in optical density fitted the Boltzmann equation,
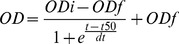
(1)Where OD is the optical density at incubation time t, OD_i_ is the OD at the initial data point, OD_f_ is the optical density at infinite time and t_50_ is the time at which the OD is equal to one-half the OD_f_.

In the rest of the assembly reaction (CA protein alone and CA in the presence of sorbitol, adonitol, maltose, arabinose, mannose, trehalose or Tyr) the time-dependent increase in optical density fitted to a linear equation. In these reactions, t_50_ values cannot be calculated but rather estimated. For all assembly reactions, the linear rate of assembly (the average increase in optical density per minute) was obtained from the slopes of the linear fit of the approximate linear part of the assembly curves.

### Dynamic light scattering measurements

For dynamic light-scattering measurements, CA was assembled at 38 µM in 50 mM Na_2_HPO_4_ (pH 8.0) buffer at final NaCl concentration of 1.5 M, either in absence or presence of 0.5 M of different polyols (measured after 30 min incubation), sugars (measured after 5 min incubation) and methylamines (measured after 10 min incubation). Size distributions were measured using Malvern Zetasizer (Malvern Instruments, Malvern, United Kingdom).

### Transmission electron microscopy

CA was assembled as described above, either in presence or absence of 0.5 M of different polyols, sugars and methylamines. After 30 min incubation with or without the examined methylamines or 1 h incubation with or without different polyols or sugars, the polymers (5 µl) were deposited on 400 mesh copper grids covered by carbon-stabilized Formvar film (SPI supplies, West Chester, PA). After 2 min, excess fluid was removed, and the grids were negatively stained with 5 µl of 2% uranyl acetate solution for 2 min. Finally, excess fluid was removed and the grids were visualized in a JEOL 1200EX electron microscope operating at 80 kV.

### Circular dichroism (CD) spectroscopy

Far-UV CD measurements were carried out using Chirascan™ spectrometer (Applied Photophysics, UK). Thermal denaturation experiments of CA (10 µM) in 50 mM Na_2_HPO_4_ (pH 8.0) buffer were performed by measuring the ellipticity at 222 nm in absence or presence (1 M) of different chemical chaperones in 0.2 cm path length quartz cell, using a temperature scan mode. Melting data were collected at 1.0°C intervals, with 20 sec setting time and 4 sec measurement time per data point, over a range of 20–75°C. Temperature was monitored by a thermocouple in the cuvette holder block. All curves were fitted to Boltzmann equation,
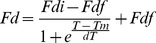
(2)Where F_d_ is the fraction of denatured protein at a given temperature T. F_d i_ is the F_d_ at the initial data point (T = 20°C), F_d f_ is F_d_ at infinite time, and T_m_ is the temperature at which the F_d_ is equal to one-half the F_d f_. For each group of chemical chaperones (polyols, sugars and methylamines) a control sample containing CA protein in the absence of chemical chaperones was measured.

### Fluorescence spectroscopy

Fluorescence spectroscopy measurements were performed using FluoroLog® Horiba Jobin Yvon FL3-11 Spectrofluorometer (Horiba Jobin Yvon Inc). Emission spectra of CA (1 µM) in 50 mM Na_2_HPO_4_ (pH 8.0) buffer were obtained using 1 cm path length cell in absence or presence of 1 M of adonitol, maltose, TMAO or choline chloride after 5 min incubation at 60°C. The experiments were carried out using an excitation wavelength of 280 nm, and 5 nm excitation or emission slits.
